# Characterization of Artificial Pneumothorax-Unrelated Pyothorax-Associated Lymphoma

**DOI:** 10.1155/2021/3869438

**Published:** 2021-01-25

**Authors:** Guang-Liang Chen, Zu-Guang Xia, Jia Jin, Bao-Hua Yu, Junning Cao

**Affiliations:** ^1^Department of Medical Oncology, Fudan University Shanghai Cancer Center, Shanghai 200032, China; ^2^Department of Oncology, Shanghai Medical College of Fudan University, Shanghai 200032, China; ^3^Department of Pathology, Fudan University Shanghai Cancer Center, Shanghai 200032, China

## Abstract

Pyothorax-associated lymphoma (PAL) is a rare disease developing from a long-term pleural cavity inflammation. Most reported PAL cases have a history of artificial pneumothorax. However, the clinical features of artificial pneumothorax-unrelated PAL remain largely unknown. Here, we reported two PAL cases diagnosed from our center in the past ten years. One case developed from asymptomatic pyothorax after pneumonectomy with a latency of 28 years, while the other case showed a relatively short latency of one year. Then we reviewed the literature of artificial pneumothorax-unrelated PAL by searching PubMed and Google Scholar from 2007. In total, nine artificial pneumothorax-unrelated PAL cases were found, predominantly in old male with median age of 76 years (ranging from 51 to 88). Most cases were diagnosed with diffuse large B-cell lymphoma (DLBCL) (*n* = 8, 88.9%) and had evidence of Epstein-Barr virus (EBV) infection (*n* = 6, 66.7%) or tuberculous pleurisy (*n* = 5, 55.6%). Notably, four cases (44.4%) had short intervals (no more than two years) between pleuritis and PAL. Regarding the overall survival, one-third cases survived more than 5 years after the diagnosis of PAL. In conclusion, the features of artificial pneumothorax-unrelated PAL are comparable with the classic type of PAL, except for some patients with short duration of pleuritis, and need to be identified. Treatment guideline of DLBCL is recommended for the management of PAL.

## 1. Introduction

Pyothorax-associated lymphoma (PAL) is a rare but distinct type of non-Hodgkin's lymphoma [1–4]. Diagnosis of PAL can be established once lymphoma cells were found in the longstanding inflammatory pleural cavity, such as chronic pyothorax [4–6]. In the recent WHO classification, PAL is defined as the prototype of diffuse large B-cell lymphoma associated with chronic inflammation (DLBCL-CI), showing a strong association with Epstein-Barr virus (EBV) infection [7–11]. Most cases of PAL are of B-cell lineage, while others show a T-cell or dual B- and T-cell immunophenotype [12–14]. In 1987, three cases of pleural B-cell lymphoma were first reported by Japanese investigators with a history of artificial pneumothorax for the treatment of tuberculous pleuritis [15]. Since then, a number of Japanese cases of PAL were published but few cases from other Asian or Western countries [2–4, 14]. The incidence of PAL is probably underestimated especially in the regions where the prevalence of pulmonary TB or EBV infection is high.

However, artificial pneumothorax has fallen into disuse due to marked progress in tuberculosis control worldwide [16]. Recently, some cases of PAL present a relatively short duration between the pleuritis and PAL rather than a very long interval that were used to be seen in the past [17–20]. Given the extremely low incidence of this disease, the clinical characteristics and management of artificial pneumothorax-unrelated PAL remain largely unknown.

In the present study, we first reported two PAL cases from our center and then summarized literatures of PAL cases without a history of artificial pneumothorax. Etiology, clinical features, differential diagnosis, and therapeutic options of PAL were discussed. We also provided some clinical recommendations for the clinical practice of PAL.

## 2. Patients and Methods

### 2.1. Diagnostic Criteria of PAL

PAL was defined as a lymphoma developing in the pleural cavity with chronic inflammation [9]. Diagnosis of PAL was established by pathological examination of biopsy specimens obtained from the lesions in the pleural cavity as previous reports [3, 4].

### 2.2. Patient Selection and Medical Record Review

We reviewed four possible cases of PAL by searching the pathology consultation specimen library of the Department of Pathology in Fudan University Shanghai Cancer Center (FUSCC) from the year of 2010 to 2019. After a thorough review of the medical history, two cases fulfilled the diagnostic criteria of PAL and were both treated in the Department of Medical Oncology, FUSCC. Clinical, laboratory, and pathological data were collected from the institutional electronic medical record system. Since there are two retrospective studies [3, 4] that have already fully addressed the clinicopathological features of artificial pneumothorax-related PAL before 2007 with a large cohort of patients, we focused on cases of artificial pneumothorax-unrelated PAL from 2007 to present. Seven PAL cases without a history of artificial pneumothorax or collapse therapy were obtained through PubMed and Google Scholar by searching the key words including pyothorax, pleural lymphoma, tuberculosis, Epstein-Barr virus, and/or rituximab. The clinical characteristics of the seven published PAL and the two cases from present study were together summarized in Table 1. International Prognostic Index (IPI) was assessed as previously described [23]. EBV infection was defined as either detectable EBV DNA or positive antibodies in sera, or EBV-encoded small RNA- (EBER-) 1/2 in tumor cells [24].

## 3. Results

### 3.1. Two Case Reports of PAL

**Figure 1:**
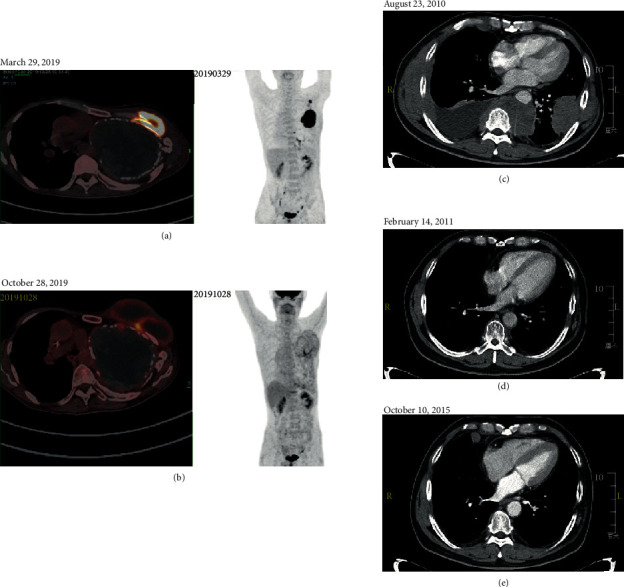
Imaging assessment of patients with artificial pneumothorax-unrelated pyothorax-associated lymphoma (PAL). Positron Emission tomography/computed tomography (PET/CT) scan in patient 1 (a-b) and contrast-enhanced chest CT  scan in patient 2 (c–e) were performed at the indicated time points.

**Figure 2:**
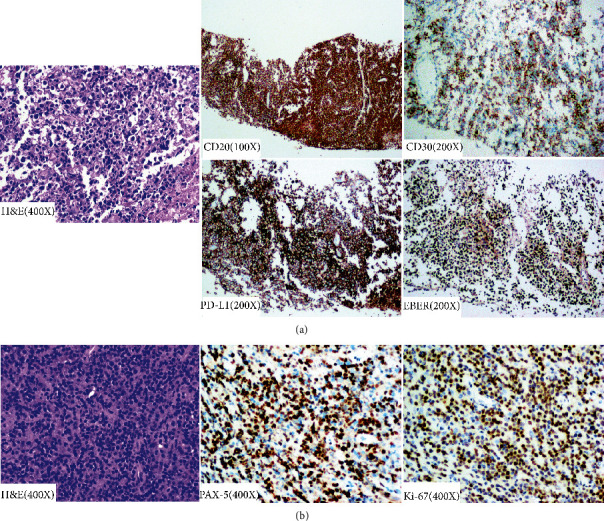
Immunohistochemical study of biopsy samples from patient 1 (a) and patient 2 (b). H&E: haematoxylin and eosin; PD-L1: programmed death-ligand 1; EBER: Epstein–Barr encoded RNA; and PAX5: paired box gene 5.

Patient 1 was a 51-year-old male, who presented with a history of fever, night sweats, pain, and a lump in the left upper chest for one month in February 2019. He previously had a left pneumonectomy for emphysematous bullae in the age of 23. Computed tomography (CT) before admission showed left lung resection, compensatory right lung hyperinflation, and a mass on the left chest wall. Positron emission tomography (PET) showed increased fluorodeoxyglucose (FDG) accumulation in the left chest wall and left axillary and thoracic aortic lymph node (Figure 1(a)). In March 2019, the patient was transferred to our hospital. Immunohistochemistry of intrathoracic biopsy revealed that the tumor cells were positive for CD20, CD30, c-Myc, BCL-2, Mum-1, and PD-L1 but negative for CD3, CD5, CD10, BCL-6, PD-1, and cyclin D1, conforming to the pathological diagnosis of diffuse large B-cell lymphoma (DLBCL) (Figure 2(a))). The Ki-67 index of tumor cells was high (80–90%). In situ hybridization using an EBER-1 probe did reveal positive signals in the nucleus of tumor cells. The EBV titer in serum was 27200 copies/ml. The level of serum lactate dehydrogenase (LDH) was 604 U/L. Thus, a diagnosis of stage II pyothorax-associated DLBCL with two points for IPI scores was determined. After one cycle of R-CHOP-21 chemotherapy (rituximab, cyclophosphamide, doxorubicin, vincristine, and prednisolone of the 21-day cycle), however, several high-grade adverse events (Grade IV, febrile neutropenia and thrombocytopenia) were observed, and a 20% reduction in chemotherapy doses was required since the second cycle of R-CHOP onward. PET-CT showed partial metabolic response after 6 cycles of treatment (Figure 1(b)), with a cystic mass on the left chest wall. Brown liquid of 200 ml was aspirated from the cystic mass, with a leukocyte count of 10823/*µ*l, platelet count 30000/*µ*l, LDH 2106 IU/l, and a few of atypical cells and phagocytic cells. The EBV titer in serum decreased to 3050 copies/ml. After multidisciplinary discussion, he received radiation therapy, with a total dose of 39.6 Gy in 23 fractions, to the local tumor site of the left chest at the end of February 2020. However, a palpable cystic lump remained on the chest after radiotherapy.

Patient 2 was a 57-year-old male, who came to our hospital with a history of left chest pain, fever, and night sweats for 1 month in July 2010. He was previously diagnosed with pyothorax and pulmonary tuberculosis one and two years ago, respectively. He also had a history of nuclear radiation exposure during military service at age 27. PET-CT scans before admission showed multiple nodules with a high level of FDG uptake in bilateral pleural. Chest CT after admission showed a shadow in the left lower lobe and posterior mediastinum, involving the chest wall and pleura, with right pleural effusion, atelectasis, and enlarged lymph nodes (Figure 1(c)). Immunohistochemical analysis of the left pleural biopsy specimens suggested DLBCL (Figure 2(b)), where the tumor cells were positive for CD20, PAX5, and BCL-2 but negative for CD10, BCL-6, Mum-1, and LMP-1. The proliferation rate (Ki-67 index) was high (80–90%). The level of serum LDH was normal. A final diagnosis was made as a stage IV pyothorax-associated DLBCL, with two points for IPI score. In August 2010, the R-CEOP-21 regimen (rituximab, cyclophosphamide, epirubicin, vincristine, and prednisolone of the 21-day cycle) was initiated. A complete response was achieved after four cycles of chemotherapy (Figure 1(d)). However, the patient was reluctant to continue with immunochemotherapy and changed to traditional Chinese medicine instead. In July 2012, chest CT showed multiple new lesions in the right lung and pleural cavity, supporting the diagnosis of relapsed DLBCL. The patient received R-ESHAP regimen (rituximab, etoposide, methylprednisolone, cytarabine, and cisplatin) as salvage therapy, with a 75% doses reduction. Though the disease obtained a complete remission again after three cycles, the patient was not eligible for stem cell transplantation because of cardiac dysfunction. However, the disease progressed again in January 2014. R-GemOx regimen (rituximab, gemcitabine, and oxaliplatin) was selected as a third-line treatment. A complete response was observed at the end of four cycles of chemotherapy. At the next onset of disease progression (Figure 1(e)), we prescribed R2 regimen (rituximab and lenalidomide) as a fourth-line treatment. Unfortunately, the patient died of a sudden cardiac disease during the maintenance treatment with lenalidomide in May 2016.

### 3.2. Clinical Characteristics of Artificial Pneumothorax-Unrelated PAL

**Figure 3:**
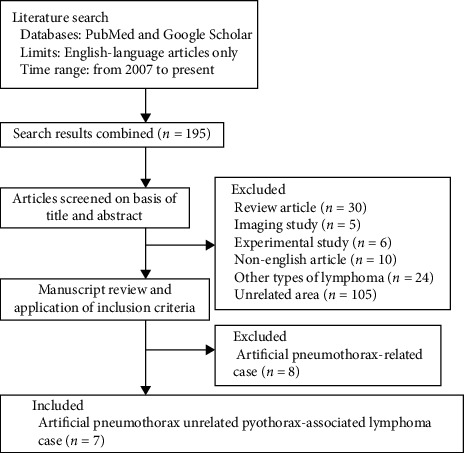
A flowchart for the search strategy and report inclusion of artificial pneumothorax-unrelated pyothorax-associated lymphoma case from PubMed and Google Scholar.

To further investigate the clinical features of artificial pneumothorax-unrelated PAL, we reviewed seven PAL cases selected from published articles (Figure 3). Results of a pool analysis of nine PAL cases (including two cases from our center) were shown in Table 1. There were seven males and two females, and the median age was 76 years (range: 51–88). Five patients (55.6%) had a history of pulmonary tuberculosis. The median interval between diagnosis of pleuritis and PAL was 27 years (range: 0–33). Interestingly, four cases (44.4%) had short intervals (less than or equal to two years). Most patients (*n* = 8, 88.9%) exhibited typical histological immunophenotype of DLBCL except one case with a dual positive for CD3 and CD20 in the recurrent lesion. Six patients (66.7%) had evidence of the EBV infection. In addition, upfront CHOP-based chemotherapy regimens were adopted in six cases, in which additional rituximab was used in four of them. Regarding survival, one-third cases survived more than 5 years after the initial diagnosis of PAL.

## 4. Discussion

In the current study, we presented two PAL cases together with seven published cases from the literature that were all artificial pneumothorax unrelated. Seven of nine patients were male, and the median age was 76 years. Most of the patients (8/9) were diagnosed with DLBCL. Tuberculosis remains the most common predisposing factor for PAL, with 55.6% of cases showing association with tuberculosis here. In addition, PAL can develop from any type of empyema, including posttraumatic empyema [8], nonspecific pleuritis [22], and pneumonectomy [20, 25].

The clinical features of PAL cases in this study were comparable with artificial pneumothorax-related PAL except for some cases having relatively short history of pleuritis. The duration of the interval between artificial pneumothorax or pleuritis and PAL is commonly decades [3, 4]. Five patients in this study had typical long duration from pyothorax to PAL, with median of 30 years, while the other four patients had short intervals of less than two years. The mechanism of subacute onset of PAL is unknown. For the cell-of-origin as determined by the Hans algorithm, the nongerminal center B-cell like (non-GCB) subtype is more prevalent in PAL with a subtype of DLBCL, which was supported by the present two cases and the case reported by Wang et al. [19]. In addition, PALs showed increased expression of activated B-cell like signature genes by quantitative polymerase chain reaction (PCR) analysis [26]. Furthermore, sequencing of the immunoglobulin heavy-chain variable (*IgV*_*H*_) gene in cell lines and clinical samples indicated the PAL cells to be derived from postgerminal center B cells [27].

It was proposed that EBV infection could contribute to the development of PAL [3]. Actually, consistent with others, we found around 70% of artificial pneumothorax-unrelated PAL cases have evidence of EBV infection [3]. However, the mechanisms underlying, which drive chronic inflammation to overt PAL, still remain elusive. Regarding the genomic landscapes, recurrent *TP53*, *Ataxia telangiectasia mutated (ATM)*, and *RAD3-related (ATR)* mutations, together with the inactivation of p16^INK4a^/Rb pathway, may induce genome instability and finally lead to malignant transformation [28, 29]. Additionally, *MYC* amplification occurred in 80% of PAL cases [30], indicating that PAL is a refractory disease. Moreover, an immunosuppressive tumor microenvironment, with tons of interleukin 6 (IL-6), IL-10, EBV-encoded microRNAs, and C-C motif chemokine receptor 4 (CCR-4)+ regulatory T-cells (Treg), may aid lymphoma cell survival in immunocompetent individuals [10, 31]. Interestingly, EB virus can induce high level of programmed cell death 1 ligand 1 (PD-L1) expression in B lymphoma cells, which may also attribute to tumor immunosuppression by PD-1/PD-L1 axis [24]. Importantly, tuberculosis [32] and other chronic inflammatory disorders [33] might induce a *T* helper (Th) 17 and Th1 cell inflammation and low antigen-specific T-cells' response in the pulmonary and lymphoid compartments. Nevertheless, the mechanism of EBV-negative PAL remains unclear.

Differential diagnosis needs to be made between PAL and primary effusion lymphoma (PEL) because both present with pleural effusion [6, 34]. PAL usually presents with a relative localized tumor mass, while PEL patients typically present with effusion in the absence of lymphadenopathy or organomegaly. PEL typically affects immunocompromised individuals with human immunodeficiency viruses (HIV) infection, while PAL does not [35]. Furthermore, human herpes virus type 8 (HHV-8) is commonly positive for PEL but negative for PAL. However, the coinfection of EBV and HHV-8 was seen in PAL cases as well [36, 37], suggesting possible pathogenic links between PAL and PEL [38]. A limitation of this study is the absence of testing for HHV-8 for two cases from our center, although clinical and pathological features supported the diagnosis of PAL.

Due to limited cases studied and lack of randomized clinical trial, optimal therapeutic strategies for PAL remain largely unrevealed, and current guidelines' recommendation primarily depends on the treatment for DLBCL. Immunochemotherapy, containing rituximab and CHOP-based regimen, is recommended for the first-line treatment of PAL. Considering the advanced age and poor performance status of most patients, dose reduction is usually required, which may further affect treatment efficiency. In addition, EBV-positive PAL showed less drug response and more unfavorable outcomes [39]. For relapsed or refractory PAL patients, aggressive salvage chemotherapy followed by autologous stem cell transplantation is recommended; however, this may not be suitable for old or fragile patients. Thus, less aggressive regimens, e.g., GDP (gemcitabine, dexamethasone, and cisplatin), plus rituximab can be considered then. In addition, lenalidomide is validated as an effective and tolerant treatment option, especially for nongerminal center B-cell (non-GCB) DLBCL, by functioning as immunomodulators [40, 41]. Lenalidomide combined with rituximab (R2) showed synergistic effect with 28–41.2% overall response rate (ORR) in R/R DLBCL [42].

In a phase II randomized trial comparing polatuzumab vedotin (Pola) [43], an anti-CD79b antibody drug conjugate, in combination with bendamustine (B) and rituximab(R) versus BR for R/R DLBCL, the overall response rates were 45% and 17.5%, and the complete response rates were 40% and 15%, respectively. The median overall survival was 11.8 months in Pola-BR group and 4.7 months in BR group. The toxicity profile of Pola-BR was acceptable. Thus, Pola-BR regimen has been approved by FDA for the treatment of R/R DLBCL after at least two prior therapies. The efficacy of immune checkpoint inhibitors, anti-PD-1 or PD-L1 monoclonal antibodies, had been evaluated for DLBCL as well. Because of low expression of PD-L1 in DLBCL tumor cells, Nivolumab [44] (anti-PD-1 monoclonal antibody) treatment resulted in only 10% response rate for R/R DLBCL. Nevertheless, the immune checkpoint inhibitors [45] might be a promising approach for EBV-positive DLBCL, as well as for PAL.

## 5. Conclusions

In conclusion, the results of this study indicate that the clinical features of PAL patients are comparable to artificial pneumothorax-related PAL, and long-term chronic pyothorax accompanied with EBV infection both contribute to the development of PAL. The etiology of PAL with short duration of pleuritis is unknown, where additional research is warranted. Attention needs to be paid to accurize the diagnosis of this rare lymphoma entity, and efforts need to be made to explore more effective and well-tolerant therapeutic regimens, such as immune checkpoint inhibitors and chimeric antigen receptor *T* (CAR-T) cell therapy.

### 5.1. Clinical Recommendations

- The diagnosis of pyothorax-associated lymphoma (PAL) should be considered when lymphoma develops in pleural cavity on imaging findings (e.g., extrapulmonary pleural masses), detectable masses and/or lymphadenopathy on physical findings, and one or more of the following symptoms: chest and/or back pain, fever, night sweats, or productive cough
- Individuals with PAL should be assessed for the presence of associated inflammatory conditions such as artificial pneumothorax, tuberculosis or tuberculous pleurisy, posttraumatic empyema, pneumonectomy, and nonspecific pleuritis
- CD30, programmed cell death 1 ligand 1 (PD-L1), Epstein–Barr virus (EBV), EBV DNA load, and human herpes virus type 8 (HHV-8) (blood or paraffin section) should be tested for patients with a clinical diagnosis of PAL
- Immunochemotherapy containing rituximab is recommended for the first-line treatment of CD20-positive PAL
- Radiotherapy should be scheduled to patients with PAL for residual tumor after first-line chemotherapy
- The immune checkpoint inhibitors, immunomodulators, or chimeric antigen receptor *T* (CAR-T) cell therapy may be considered for patients with relapsed or refractory PAL

## Figures and Tables

**Table 1:** Characteristics of artificial pneumothorax-unrelated pyothorax-associated lymphoma.

Investigator	Gender	Age	Latency	Cause of empyema	EBV	Lymphoma subtype	Regimen	Overall survival	Reported
Moriya et al. [21]	M	64	30 yrs.	Empyema	—	DLBCL	Decortication	>5 yrs.	2010
Tzeng et al. [17]	M	79	0.7 yrs.	Tuberculosis	n.a.	DLBCL	CT	n.a.	2010
Terada [22]	F	88	0.7 yrs.	Nonspecific pleuritis	+	DLBCL	n.a.	n.a.	2012
Taniguchi et al. [8]	F	78	33 yrs.	Trauma	+	CD3+ and CD20+ lymphoma	R–CHOP	>9 yrs.	2015
Yun et al. [16]	M	60	30 yrs.	Tuberculosis	+	DLBCL	Surgery + CT	n.a.	2015
Hibino et al. [18]	M	81	33 yrs.	Tuberculosis	+	DLBCL	CHOP	>0.3 yrs.	2015
Wang et al. [19]	M	76	0	Tuberculosis	+	DLBCL, non-GCB	R–CVP	>1.5 yrs.	2019
This study	M	51	27 yrs.	Pneumonectomy	+	DLBCL, non-GCB	R–CHOP + RT	>1 yr.	2021
This study	M	57	2 yrs.	Tuberculosis	—	DLBCL, non-GCB	R–CHOP	4.8 yrs.	2021

*Note.* n.a.: not available.; RT: radiotherapy; DCBCL: diffuse large B-cell lymphoma; EBV: Epstein-Barr virus; yrs.: years; CT: chemotherapy; and GCB: germinal center B-cell like.

## Data Availability

Data sharing is not applicable to this article, as no datasets were generated or analyzed during the current study.
